# A Cell-type-resolved Liver Proteome*[Fn FN2]

**DOI:** 10.1074/mcp.M116.060145

**Published:** 2016-08-25

**Authors:** Chen Ding, Yanyan Li, Feifei Guo, Ying Jiang, Wantao Ying, Dong Li, Dong Yang, Xia Xia, Wanlin Liu, Yan Zhao, Yangzhige He, Xianyu Li, Wei Sun, Qiongming Liu, Lei Song, Bei Zhen, Pumin Zhang, Xiaohong Qian, Jun Qin, Fuchu He

**Affiliations:** From the ‡State Key Laboratory of Proteomics, Beijing Proteome Research Center, Beijing Institute of Radiation Medicine, Beijing 100039, China;; §National Center for Protein Sciences (The PHOENIX center, Beijing), Beijing 102206, China;; ¶School of Life Sciences, Tsinghua University, Beijing 100084, China;; ‖Alkek Center for Molecular Discovery, Verna and Marrs McLean Department of Biochemistry and Molecular Biology, Department of Molecular and Cellular Biology, Baylor College of Medicine, Houston, Texas 77030;; **State Key Laboratory of Genetic Engineering and Collaborative Innovation Center for Genetics and Development, School of Life Sciences, Institute of Biomedical Sciences, Fudan University, Shanghai 200433, China

## Abstract

Parenchymatous organs consist of multiple cell types, primarily defined as parenchymal cells (PCs) and nonparenchymal cells (NPCs). The cellular characteristics of these organs are not well understood. Proteomic studies facilitate the resolution of the molecular details of different cell types in organs. These studies have significantly extended our knowledge about organogenesis and organ cellular composition. Here, we present an atlas of the cell-type-resolved liver proteome. In-depth proteomics identified 6000 to 8000 gene products (GPs) for each cell type and a total of 10,075 GPs for four cell types. This data set revealed features of the cellular composition of the liver: (1) hepatocytes (PCs) express the least GPs, have a unique but highly homogenous proteome pattern, and execute fundamental liver functions; (2) the division of labor among PCs and NPCs follows a model in which PCs make the main components of pathways, but NPCs trigger the pathways; and (3) crosstalk among NPCs and PCs maintains the PC phenotype. This study presents the liver proteome at cell resolution, serving as a research model for dissecting the cell type constitution and organ features at the molecular level.

Organs consist of multiple cell types that are arranged with a high level of organization. The architecture and interactions between the different cell types define the identity and microenvironment of the organ. Generally, parenchymal cells (PCs)^1^ and many different types of nonparenchymal cells (NPCs) play significant roles in the organ. PCs are the most abundant cell type, performing the dominant roles of the organ. NPCs usually account for a minor portion of the cellular population, regulating the functions and microenvironment of the organ. The material exchanges, ligand-receptor recognition, signal transduction, and pathway crosstalk among cell types, especially between PCs and NPCs, are critical for performing organ functions and maintenance. In this process, the patterns of protein expression in different cell types undertake fundamental tasks. Thus, a proteome map of an organ with cell type resolution would enable us to dissect the basic features of the cellular composition of the organ. However, despite extensive studies focused on function and regulation between different cell types, because of the lack of a global view at the “-omics” scale, the features and mechanisms of the cellular composition of organs are still unknown.

As the largest solid organ in the body, the liver consists of multiple cell types that are responsible for the organism-level functions of metabolism, detoxification, coagulation, and immune response. Four major liver cell types—hepatocytes (HCs), hepatic stellate cells (HSCs), Kupffer cells (KCs), and liver sinusoidal endothelial cells (LSECs)—spatiotemporally cooperate to shape and maintain liver functions. HCs constitute ∼70% of the total liver cell population. The remaining population is composed of the NPCs, namely LSECs, KCs and HSCs (1). As the parenchymal portion of the liver, HCs are primarily engaged in the basic functions of the liver, including lipid metabolism, drug metabolism, and the secretion of coagulation and complement factors (2). KCs, which represent one-third of the NPCs in the liver (3), serve as immune sentinels. Although HSCs comprise only 5% of the liver cells, they play central roles in vitamin A and lipid storage (4, 5). LSECs, which comprise the largest part (50%) of liver NPCs, separate the underlying HCs from the sinusoidal lumen (6).

The distinct cell types of the liver are arranged in a highly organized architectural pattern with individual cells in communication with each other (7). Correlation and crosstalk between the different cell types are common (8). It has been increasingly recognized that under both physiological and pathological conditions, HCs are regulated by factors released from neighboring NPCs (9). KCs, in response to pathogenic agents, produce inflammatory cytokines, growth factors, and reactive oxygen species (ROS) that induce hepatic injury (10). Acute damage activates the transformation of hepatic stellate cells into myofibroblast-like cells that play a key role in the development of liver fibrosis (11). LSECs contribute to liver regeneration after liver injury (12). Although the cooperative pathways between several types of liver cells, including IL6-Jak-STAT (13), and TGFβ-SMAD (14), have been studied, the global network of the different cell types has not been previously reported. Therefore, the liver is an ideal model organ for studying the features and mechanisms of the cellular composition of organs. Moreover, the liver is composed of obvious PC and NPC types, which allows us to investigate the cooperation and crosstalk between these cell types.

Mass spectrometry (MS)-based proteomics is a powerful tool that provides insights into the spatiotemporal patterns of protein expression (15). The liver is the first organ whose proteome was investigated at the organ level (16), both at fetal (17) and adult stages (18). In recent years, considerable progress in MS techniques has made the precise characterization of the proteome possible. S. Babak Azimifar *et al.* reported cell type resolution liver proteome data (19), providing quantitative proteome patterns of individual cell types of the mammalian organ. In addition, this work highlighted the importance of cell type resolution proteomics in understanding liver function. However, the researchers employed a less accurate identification approach to increase the proteome coverage, which could cause confusion in data analysis and minimize the value of the cell type resolution data set. Thus, despite improvements in liver proteomics, previous studies have presented data sets that have provided little comprehensive insight into liver biology. The proteomic mechanisms involved in the division of labor and the collaboration and crosstalk between cell types have been masked and have not yet been characterized.

In this study, we chose the liver as a model organ to investigate the features and mechanisms of the cellular composition of organs by screening the cell-type-resolved liver proteome and secretome. We isolated four liver cell types with high purity and viability and employed cutting-edge MS approaches to profile the proteomes of these cell types. Comprehensive bioinformatics analysis revealed the basic features of cellular composition and liver biology associated with the different cell types, including pathway complementarity, maintenance, and crosstalk between cell types. In contrast to traditional proteomics works that merely described and presented broad-scale data, our study provides a substantial amount of novel knowledge in cellular composition of the organ based on an integrated “-omics” analysis and progressive logic.

## EXPERIMENTAL PROCEDURES

### 

#### 

##### Experimental Design and Statistical Rationale

We used three male C57BL/6J mice as a group for liver cell isolation each time, with three biological replicates. We isolated HCs, HSCs, KCs, and LSECs from livers simultaneously, with high purity and viability. RNA for each cell was extracted for Transcriptome after quality control and whole cell protein was extracted separately, followed by digestion in solution and RP-HPLC for peptide separation and LC-MS/MS for protein identification and quantification to profile the proteomes of these cell types. Comprehensive bioinformatics analysis revealed the basic features of cellular composition and liver biology associated with the different cell types, including pathway complementarity, maintenance, and crosstalk between cell types.

Mann-Whitney *U* test was applied to test whether two population means are equal; two populations includes shortest lengths of Specific TFs/Nonspecific TFs, functional category entropy of four liver cell types and so on. The enrichment of specific ontology terms (TFs, GO and KEGG) was tested using a Hypergeometric Test. For Multiple tests, Bonferroni multiple testing correction was used to control the FDR. Difference with *p* value smaller than 0.05 was considered statistically significant.

##### Reagents

The following reagents were used: collagenase type IV (Invitrogen, Carlsbad, CA), trypsin inhibitor (Amresco, Cochran Solon, OH), DNase I (AppliChem, Gatersleben, Saxony-Anhalt, Germany), bovine serum albumin (BSA, Sigma-Aldrich, Merck KGaA, Darmstadt, Germany), DMEM (Dulbecco's modified Eagle's medium, Sigma-Aldrich, Merck KGaA, Darmstadt, Germany), fetal bovine serum (FBS, Hyclone, South Logan, UT), Optiprep^TM^ density gradient liquid (Axis-shield, Rodelokka, N-0504 Oslo, Norway), ASGPR1 (Santa Cruz Biotechnology, Dallas, TX), goat anti-mouse lgG-PE (Santa Cruz Biotechnology), F4/80 (eBioscience, Santa Clara, California), CD146 (Miltenyi Biotec, Bergisch Gladbach, Germany), CD45 (Miltenyi Biotec, Bergisch Gladbach, Germany), APC Rat IgG2b k isotype (BD Pharmingen, San Jose, CA), fluorescein isothiocyanate (FITC) Rat IgG2b k isotype (BD Pharmingen), phycoerythrin (PE) Rat IgG2b k isotype (eBioscience), IC fixation buffer (eBioscience), and permeabilization buffer (eBioscience).

##### Mouse Liver Cell Isolation and Evaluation By Cell Type

Normal male C57BL/6J mice (8 weeks old, 25–28 g) were used for liver cell type isolation. Two-step liver perfusion digestion *in situ* was performed with collagenase IV and DNase I using a previously described protocol with some modifications (20). We isolated HCs, HSCs, KCs, and LSECs simultaneously using a combination of modified collagenase-based density gradient centrifugation and fluorescence-activated cell sorting (FACS) with high purity, viability, and yield. Cell purity was assessed by cytological microscopy, electron microscopy, immunocytochemistry, and flow cytometry. Cell viability was determined by 7-aminoactinomycin D (7-AAD)-stained flow cytometry, and cell yield was determined by cell count.

Below, the methods for isolating and assessing each cell type are described separately. HCs were isolated by modified *in situ* perfusion followed by natural sedimentation after differential centrifugation to enrich the HCs and then PE-conjugated ASGPR1-marked FACS to purify and sort the HCs. The sorted cells were then labeled with FITC-conjugated CD146 to evaluate cell purity. For KCs and LSECs, cells between 11.2 and 17% in the Optiprep^TM^ density gradient working solution were carefully collected. The collected cells were primarily a mixture of KCs and LSECs. We then labeled the cell mixture with phenotypic markers and purified specific cell populations by FACS. Specifically, PE-conjugated F4/80 and FITC-conjugated CD146 were used to label KCs and LSECs, respectively. The corresponding isotype antibodies were also used as negative controls to measure the nonspecific binding of the specific antibodies. After sorting, these two cell types were back-tested to determine the purity. HSCs, which were suspended in a less than 8.2% Optiprep^TM^ density gradient working solution, were removed and labeled with PE-conjugated F4/80 and FITC-conjugated CD146 for FACS analysis. Forward and side scatter gates were set to exclude debris and to include all viable cells. Negative cells without positive markers of F4/80 and CD146 were sorted and back-tested to confirm the purity of HSCs. All data were acquired with a BD FACS Aria II instrument and were analyzed with Diva 6.1.2 (BD Biosciences, Franklin Lakes, NJ).

##### Cell Culture

Primary HCs, HSCs, KCs, and LSECs were cultured in DMEM supplemented with 20% FBS, penicillin/streptomycin (100 U/ml), and 2 mm glutamine at 37 °C and 5% CO_2_ in collagen-coated plates. Cells were cultured in six-well plates at a density of 5 × 10^5^ cells/ml. The state of cell culture growth was recorded in real time with inverted phase contrast microscopy.

##### Sample Preparation for RNA Sequencing and MS Analysis

A total of 1 × 10^6^ cells of the isolated primary cell types were collected for RNA or protein extraction. Total RNA was isolated from primary cell types using a Qiagen reagent kit according to the manufacturer's protocol. Proteins were extracted with 8 m urea. After protein extraction from each cell type, gel electrophoresis of the whole cell extract was performed with a 12% separating gel and a 5% stacking gel at 80 V for 20 min, followed by 120 V for 60 min. Coomassie brilliant blue staining was used to mark the protein bands in all samples. The protein sample was reduced with dithiothreitol and alkylated with iodoacetamide in the dark and then was finally digested using sequencing grade trypsin at an enzyme/protein mass ratio of 1:50 overnight at 37 °C. The reaction was stopped by the addition of 0.1% formic acid (FA).

##### Sample Preparation for Secretome Analysis

HCs and KCs were isolated and purified as described above and then plated in DMEM/1640 supplemented with 10% FBS, penicillin/streptomycin (100 U/ml) and 2 mm glutamine at 37 °C and 5% CO_2_. After the cells attached, they were washed with serum-free DMEM/1640 three times to remove FBS and cell debris and cultured with serum-free DMEM/1640 for an additional 24 h. For secretome studies, we collected the cell supernatant in a clear centrifuge tube and centrifuged at 100,000 × *g* and 4 °C for 20 min to remove cells and debris. We then transferred the supernatant to fresh centrifuge tubes, added trichloroacetic acid (TCA) to a final concentration of 12%, and incubated at 4 °C overnight to precipitate the secretory proteins. Afterward, protein precipitations were collected by centrifugation at 24,000 × *g* for 10 min. The protein pellet was resuspended and washed carefully with 1 ml of cold acetone at −20 °C twice. We then added 10 μl of 8 m urea to resolve the protein pellet and took 0.5 μl of the protein solution to measure the protein concentration. We took 30 μg of protein for the proteome analysis. The secreted proteins were digested with trypsin (1:50) overnight at 37 °C. The digestion process was ended by the addition of 0.1% FA. The tryptic peptides were separated and identified by RP-HPLC (reversed-phase high-performance liquid chromatography) and liquid chromatography tandem mass spectrometry (LC-MS/MS) as described by Ding *et al.* (21). The secretomes of HCs and KCs were analyzed independently in three biological replicates.

##### Two-dimensional RP LC-MS

To perform an in-depth proteome screening, dual short-gradient two-dimensional reversed-phase liquid chromatography mass spectrometry (2D-RPLC-MS) (21) was performed for the four liver cell types. Briefly, 200 μg of total tryptic peptides was separated into 24 fractions with high-pH RPLC (Durashell RP column 5 μm, 150 Å, 250 mm × 4.6 mm i.d., Agela; mobile phase A (2% acetonitrile, pH = 10.0) and B (98% acetonitrile, pH = 10.0)). The eluent samples were dried and reconstituted in HPLC loading buffer (0.1% (v/v) FA, 2% (v/v) acetonitrile in water), and 24 fractions were submitted to low-pH RPLC-MS (C18 column, 3 μm C18) for identification. Mobile phase A consisted of 0.1% FA in water, and mobile phase B consisted of 0.1% FA in acetonitrile. The Orbitrap Q-Exactive source MS was operated at 1.8 kV. For full MS survey scans, the automatic gain control (AGC) target was 3e6 and the scan range was from 300 to 1400 m/z, with a resolution of 70,000. The 75 most intense peaks with charge states of 2 or above were selected for fragmentation via higher-energy collision dissociation (HCD) with a normalized collision energy of 27%. The dynamic exclusion time for MS/MS was set as 18 s. The MS2 spectra were acquired with a resolution of 17,500.

##### Parallel Reaction Monitoring (PRM) Analysis

HCs and NPCs from three wild-type C57BL/6J mice livers were prepared separately via the gravity centrifugation method (HC, centrifugation at 50 × *g*; NPC, centrifugation at 600 × *g*). Cell pellets of six samples, HC1/NPC1 (mouse 1), HC2/NPC2 (mouse 2) and HC3/NPC3 (mouse 3) were suspended in lysis buffer (8 m urea containing 1% phenylmethylsulfonyl fluoride (PMSF)) and sonicated using twenty 0.2-s pulses with 1-s intervals for cooling between each pulse. The extracted proteins were reduced at 37 °C for 4 h and alkylated at room temperature in the dark for 45 min by the addition of dithiothreitol (at a final concentration of 10 mm) and iodoacetamide (at a final concentration of 25 mm). Sequencing grade trypsin (Promega, Madison, WI) was added to each sample at a 1:50 enzyme/substrate ratio, and the reactions were incubated overnight at 37 °C. The digestion mixtures were separated on 4.6 × 250 mm XBridge BEH300 C18 column (Waters) at a flow rate of 0.7 ml/min using the following linear gradient: 5–35% phase B for 30 min (phase A: 2% acetonitrile (ACN) in ammonium hydroxide solution, pH 10; phase B: 98% ACN in ammonium hydroxide solution, pH 10; column temperature, 45 °C), 35–95% phase B for 2 min, 95% phase B for 5 min, 95–5% phase B for 2 min, 5% phase B for 6 min. The eluate was collected each minute into vials starting at the sixth minute. Vials 6, 18, and 30 were pooled, with a total of 12 fractions prepared by the leaping pooling strategy.

Proteopeptides of target gene products (GPs) were identified and focused in the exact RT windows and fractions by data dependent acquisition (DDA) scan. The parent ions in the table were monitored in the different fractions of 6 samples on an Easy nLC system (Thermo Fisher Scientific) coupled with Fusion (Thermo Fisher Scientific). Peptides were separated on a homemade reverse-phase capillary column (75 μm × 150 mm, New Objective) packed with C18 media (Agela, 3 μm, China) using the following gradient: 5–8% phase B (98% ACN in 0.1% formic acid) for 8 min, 8–22% phase B for 50 min, 22–32% phase B for 12 min, 32–90% phase B for 1 min, and 90% phase B for 7 min at a flow rate of 350 nL/min. The peptides were analyzed using full scan plus PRM modes. The full mass within the range of 300 to 1400 *m*/*z* was collected. The MS1 resolution was set at 30,000. For PRM spectra acquisition, the resolution parameter was 30,000, the HCD collision energy was 32%, the AGC target value was 1.0e5 and the maximum IT time was 64 ms. All of the raw files were processed using Skyline 3.1. The intensities of three fragment ions were summed for peptide quantification. The intensities of up to three peptides were summed and used for GP quantitative comparison.

##### Protein Identification and Quantification

Raw files from Orbitrap Q-Exactive were searched with the MASCOT 2.3 search engine with percolator against the mouse RefSeq protein database (29,764 proteins, updated on 07–01-2013) in the Proteome Discoverer (Version 1.4). A target-decoy-based strategy was applied to control both peptide- and protein-level false discovery rates (FDRs) lower than 1% (22). Two of missed and/or nonspecific cleavages were permitted. The fixed modification was carbamidomethyl (C) and the variable modifications were oxidation (M) and acetyl (Protein N-term). Mass tolerance for precursor ions was 20 ppm, and 50 mmu for fragment ions. Proteome quantification was performed as previously reported (21) with the iBAQ algorithm (23) and then normalized to FOT (a fraction of the total protein iBAQ amount per experiment).

##### Bioinformatics Analyses for the Expression Profiles

Gene Ontology (GO) assignments were made using the Mouse Genome Informatics (MGI) database (downloaded on 15 May 2016). Pathway assignment was performed using the Kyoto Encyclopedia of Genes and Genomes (KEGG) data set (Release 53.0). The enrichment of specific GO terms was tested using a hypergeometry test, followed by the Bonferroni multiple testing correction to control for the FDR. The function category entropy of a protein set *S* was calculated as −Σ*F_i_* log *F_i_*, where *F_i_* is the frequency of a function category *i* in *S,* which can be computed using the equation: *F_i_* = *T_i_*/Σ*_i_^n^T_i_*, where *T_i_* is the number of proteins of *S* in category *i* and *n* is the total number of distinct functional categories in *S*. Both the GO biological process and KEGG pathway categories were used to calculate the functional category entropy. The proteomap and transcriptomap were plotted using the web service http://bionic-vis.biologie.uni-greifswald.de/.

##### Crosstalk Network Among Four Liver Cell Types

The ligand-receptor interactions were downloaded from DLRP (24), IUPHAR (25), and the literature. Only the interactions between secreted ligands (enhanced proteins or proteins secreted by the four cell types) and expressed receptors with active downstream KEGG (26) signaling pathways were selected (27). Cell-enhanced proteins were identified using the ratio of a protein in a particular cell to the average level in all four types of liver cells (at least 2-fold) (28), and active signaling pathways were defined as those with significantly enriched enhanced constituent proteins (hypergeometric test, *p* value<0.05; ligand-receptor-transcription factors (TFs)-target genes) and when all of the pathway nodes could be detected in the proteomic or transcriptomic profile. TFs and their target gene information were obtained from CellNet (29). Mouse genes were mapped to human orthologs using the MGI database.

## RESULTS

### 

#### 

##### Proteome Profiling of Four Major Types of Liver Cells

**Fig. 1.**
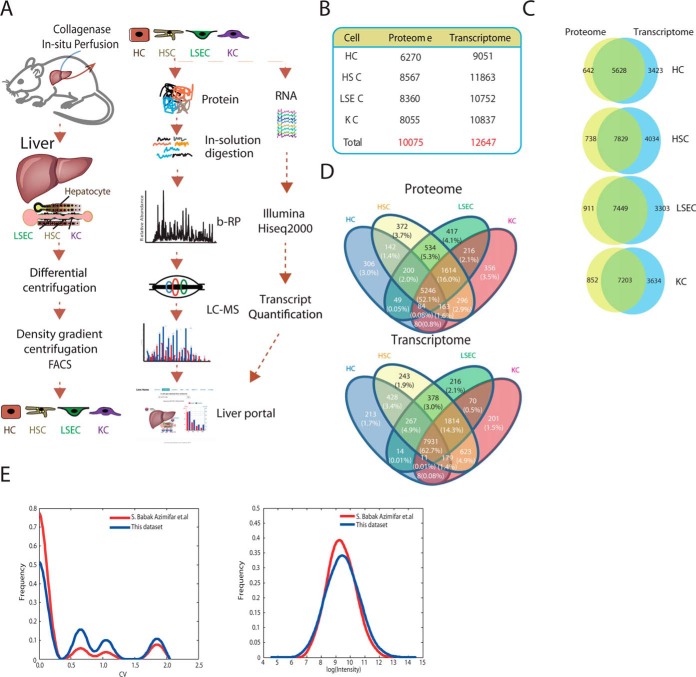
**Proteomes and transcriptomes of four major liver cell types.** *A*, Schematic illustration of the experimental workflow. Four major liver cell types—HCs, HSCs, KCs, and LSECs—were isolated from mouse liver with two-step collagenase perfusion. Proteins from whole-cell extracts and culture supernatants were collected and submitted to an MS platform. Protein samples were fractionated, digested, and analyzed on a high-resolution Orbitrap mass spectrometer. Tandem MS data were searched against a mouse RefSeq database using the MASCOT engine. An aliquot of the cell pellet was submitted for RNA-Seq. *B*, Identification of GP numbers at the protein and mRNA levels for four liver cell types. *C*, Venn diagram of the identified GP numbers at the protein and mRNA levels among the four liver cell types. *D*, Venn diagram of expressed genes at the mRNA and protein levels among the four liver cell types. *E*, Coefficients of variation of proteins expressed across four cell types and dynamic ranges of the four cell type proteomes in this data set, in comparison with a previous report.

To generate a cell type resolution liver proteome, we isolated four major types of liver cells (HCs, HSCs, KCs, and LSECs) using a modified protocol including two-step collagenase perfusion, centrifugation, and FACS (30) (Fig. 1*A*). With the modified method for cell isolation and validation, the cell yields of HCs, HSCs, KCs, and LSECs were approximately (7.0 ± 0.4) × 10^7^, (1.1 ± 0.2) × 10^6^, (2.1 ± 0.2) × 10^6^, and (2.1 ± 0.2) × 10^6^ per mouse, respectively. The viabilities of HCs, HSCs, KCs, and LSECs evaluated with trypan blue staining and 7-AAD flow cytometry were (90.6 ± 0.7)%, (88.3 ± 0.5)%, (88.4 ± 0.5)%, and (87.3 ± 0.3)%, respectively. A variety of evidence obtained by bright field microscopy, electron microscopy, autofluorescence tests, immunocytochemistry, and FACS analysis confirmed that the purities of HCs, HSCs, KCs, and LSECs were (98.6 ± 0.5)%, (93.7 ± 0.4)%, (94.6 ± 0.2)%, and (98.0 ± 0.5)%, respectively (supplemental Fig. S1*A*–S1*D*). We also assessed the quality of the proteins extracted from the four cell types by SDS-PAGE to ensure that protein extraction was complete and resulted in high-quality protein without degradation (supplemental Fig. S1*E*). Isolated primary cells were cultured in serum-free medium for secretome identification.

We employed the Fast-seq (21) approach that we previously developed for proteome and secretome identification, and biological triplicates of the four liver cells yielded protein identifications in the range of 6200 to 8500 GPs from single cell types and an overall total of 10,075 GPs from the four cell types (Fig. 1*B*, supplemental Table S1, and supplemental Table S6). We identified around 2000 GPs from the combined secretome of the HCs and KCs (1149 GPs in HCs and 1420 GPs in KCs, respectively), of which ∼1000 GPs (574 GPs in the secretome of HCs and 738 GPs in secretome of KCs, respectively) were located in the extracellular region (GO: 0005576 Extracellular Region) (supplemental Table S1). We also identified a total of 10,616 GPs from the liver proteome (proteome of the four cell types of the liver) and secretome. As evidence of good reproducibility, we found high correlations in protein abundance between the biological replicates of the same cell type (0.83–0.88) (supplemental Fig. S2*A*, supplemental Fig. S2*B*). We also found that the proteins detected in only one or two replicates had relatively high variations in expression levels in different cell types and lower abundance levels than proteins detected in all three replicates (supplemental Fig. S2*C*).

RNA-Seq profiling of the same cells identified 9000 to 11,800 protein-coding genes with more than 1 fragment per kilobase of exon per million fragments mapped (FPKM) (supplemental Table S1). Comparisons between the proteome and the transcriptome revealed high overlap of identification but modest abundance correlations between mRNA and proteins (0.53 to 0.63) (Fig. 1*B*, Fig. 1*C*, Fig. 1*D*, supplemental Fig. S2*D*, supplemental Fig. S2*E*, and supplemental Fig. S2*G*). Intriguingly, there were clear GO enrichments of over- and under-represented proteins in the detected proteome compared with the transcriptome (supplemental Table S2). Generally, over-represented proteins were enriched in mitochondrion, ribosome, and metabolic pathways, whereas under-represented proteins were enriched in the extracellular space, membrane proteins, and TFs (supplemental Fig. S2*F*, supplemental Fig. S2*I*, and supplemental Table S3). The bias between proteomic and transcriptomic data revealed biological features of proteins and transcripts. For example, some secreted proteins, identified in the secretome but not in the cell-resident proteome, were located in the extracellular space, but their corresponding transcripts were detected in the transcriptome (supplemental Fig. S2*I*). This statement is based on the genes whose proteins are in the secretome and match the transcripts in the transcriptome shown in supplemental Table S1. Thus, the enrichment of the transcriptome in the extracellular space was attenuated after we incorporated the secretome with the resident proteome in the comparison (supplemental Fig. S2*H*, supplemental Fig. S2*J* and supplemental Table S1).

We surveyed the coefficients of variation and dynamic ranges of the total identified proteins in the four cell types (Fig. 1*E*). Our data set showed greater variations and dynamic ranges of the proteome distributed in the four cell types compared with a previous report (19). The difference in the proteome diversity of the four cell types between the two data sets may be explained by differences in cell purity and the proteomics workflow. We employed a FACS approach to purify cells and achieved a purity of more than 90%, whereas the previous work used a MACS approach that typically resulted in cell populations with 80% purity or less. Furthermore, the previous study used a “match between runs” algorithm to increase proteome coverage, which likely introduces more false identifications and quantification Specifically, it employed cell lines to build up a “peptide library.” To further demonstrate the potential for inaccurate identification and quantification, we employed MaxQuant to search our MS raw files using the same parameters that were used in the previous study. As shown in supplemental Table S1, we noted 15–50% additional identifications in the four liver cell types, reaching the same level of protein identification as the previous report.

##### Proteome Features of the Four Major Liver Cell Types

**Fig. 2.**
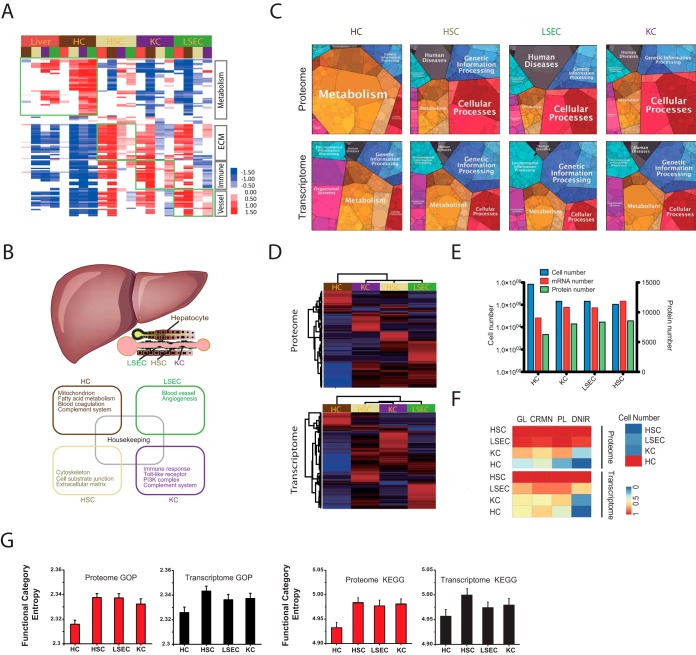
**Proteome features of the four liver cell types.** *A*, Heatmap of KEGG and PANTHER pathways shows significantly elevated (red) or decreased (blue) products in each of the four cell types compared with other cells. *B*, A proposed model illustrating liver cell type specificities. *C*, Proteomap and transcriptomap of HCs, HSCs, LSECs, and KCs. Every tile represents one type of GP. Tiles are arranged and colored according to the hierarchical GO terms. Tile sizes represent the mass fractions of GPs. *D*, Hierarchical clustering of the proteome and transcriptome of HCs, HSCs, LSECs, and KCs shows that HCs have different results at both the mRNA and proteome levels, whereas the other three liver cell types are co-clustered. *E*, The cell numbers of the four cell types are negatively related to the identified mRNA and protein coding gene numbers. *F*, The over-representation degrees of the complex genes in each cell type. GL, long genes (>28,256); CRMN, genes with multiple cis-regulatory modules (CRMN≥5); PL, genes encoding long proteins (>512); DNIR, genes encoding multi-domain proteins (domain number in proteins containing domain repeats>2). Colors ranging from blue to red represent increased gene complexity. *G*, Function category entropy of each liver cell type. Red bars represent the function category entropy of the sets of identified GPs in HCs, HSCs, LSECs, and KCs in the proteome and transcriptome. The error bars mark one standard deviation on each side of the average from 100 random samples. For transcriptomic and proteomic profiles, the functional category entropy of HC proteins is significantly lower than that of the other three cell types (*p* < 0.05, Mann-Whitney test). The biological process of Gene Ontology (“GOP”) and KEGG pathway ontology (“KEGG”) are used to compute the functional category entropy.

GO/pathway enrichment analysis of the cell-type-specific proteome revealed high consistency between proteome features and the physiological activities of each cell type (Fig. 2*A*, Fig. 2*B*, supplemental Fig. S3*C* and supplemental Table S3). The proteome and GO/pathway profile of HCs resembles that of overall liver, indicating that the biological processes of the liver are mainly performed by the parenchymal portion (Fig. 2*A* and supplemental Fig. S3*A*). The visualized proteomap (31) of both the proteome and the transcriptome of the four cell types indicates that metabolism dominates the cellular processes of HCs (Fig. 2*C*). By clustering the data sets of the four cell types, we observed a closer correlation between NPCs and the unique pattern of PCs (*i.e.* HCs) on both the proteome and the transcriptome (Fig. 2*D* and supplemental Fig. S3*A*). Interestingly, the cell populations of the four cell types in the liver were negatively correlated with the number of identified mRNAs and protein-coding genes (Fig. 2*E*). HCs, which represent the largest cell population in the liver, expressed the lowest number of genes and covered the fewest genes in almost every chromosome except the mitochondrial chromosome (chrMt) (supplemental Fig. S3*B*). In addition, HCs had the lowest gene expression complexity and functional entropy (Fig. 2*F* and Fig. 2*G*), suggesting that HCs have the highest homogeneity of gene expression and function among the four major cell types in the liver.

Mapping liver disease-related genes to the proteomes of the four cell types revealed that liver disease-related proteins are more enriched in NPCs than in PCs. For example, genes related to autoimmune hepatitis (AIH) are highly enriched in KCs and HSCs. Genes related to liver fibrosis and nonalcoholic steatohepatitis (NASH) disease were over-represented in HSCs (supplemental Fig. S3*D* and supplemental Table S3). The enrichment of liver disease-related genes in normal NPCs suggests that liver diseases might be caused by dysregulation of the NPC compartment and indicates that the NPC compartment could play an important role in the regulation of normal PCs.

##### The Division of Labor and Functional Cooperation Among the Four Cell Types: PCs Produce Downstream Pathway Components, but NPCs Trigger Pathways

**Fig. 3.**
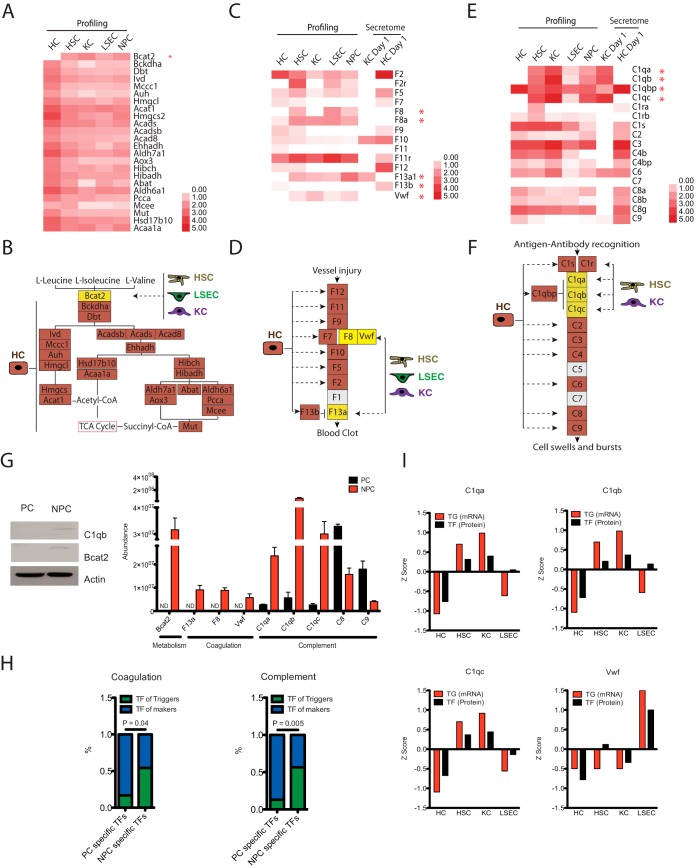
**Divisions of labor of the four liver cell types.** *A*, Protein expression patterns of the coagulation pathway in the proteomes and secretomes of the four cell types. *B*, Complementarity of coagulation component expression in HCs, HSCs, KCs, and LSECs. *C*, Protein expression patterns of the complement pathway in the proteomes and secretomes of the four cell types. *D*, Complementarity of complement component expression in HCs, HSCs, KCs, and LSECs. *E*, Protein expression patterns of the valine/leucine/isoleucine metabolism pathway in the proteomes of the four cell types. *F*, Complementarity of valine/leucine/isoleucine metabolic component expression in HCs, HSCs, KCs, and LSECs. *G*, WB and PRM validation of cell-type-specific proteins. Equal amounts of proteins from PCs and NPCs were loaded. ND, not detected. *H*, Enrichment of triggers and downstream pathway components in the target gene groups of specific TFs of PCs and NPCs. *I*, Z scores of C1qa, C1qb, C1qc, and Vwf mRNA expression and their upstream TF protein expression in the four cell types.

The analysis of the proteomes in different liver cell types allows us to investigate the basic roles of the four cell types in the context of the whole organ. Metabolism, complement, and coagulation cascades are three systemic pathways primarily executed by the liver. Intriguingly, we found obvious functional complementarities in these three biological processes among the four cell types. Bcat2 catalyzes the first reaction in the catabolism of essential branched-chain amino acids, triggering the leucine/isoleucine/valine metabolism cascade. HCs expressed almost all of the essential catalytic enzymes, except the first one, Bcat2. A high level of Bcat2 expression was identified in NPCs (Fig. 3*A* and Fig. 3*B*). The liver synthesizes and secretes 80 to 90% of the complement and coagulation proteins in the body (32, 33). In our study, the complement and coagulation cascades ranked at the top of the enriched pathway in the HC secretome. The gene expression patterns of the complement and coagulation cascades in different liver cell types revealed similar complementarity with respect to metabolism. HCs extensively expressed the majority of coagulation components, such as factors XII, XI, IX, X, VII, V, and II, but they expressed no or very low amounts of “triggers,” such as factors VIII, Vwf, and XIIIa (Fig. 3*C* and Fig. 3*D*). Factor XIIIb, an inhibitor of XIIIa, was exclusively identified in HCs. In contrast, HSCs, KCs, and LSECs expressed the essential trigger factors VIII, Vwf, and XIIIa and did not express the inhibitor XIIIb. Similarly, HCs expressed high levels of C2, C3, C4, C6, C8, and C9 and low levels of the complement triggers C1qa/b/c. In HCs, an abundance of the C1q complex inhibitor C1qbp was identified. Conversely, HSCs and KCs expressed active components of the C1q complex (Fig. 3*E* and Fig. 3*F*).

The complementarity of liver cell types in the metabolism, complement and coagulation pathways indicated that the role of PCs is to make pathway enzymes but that the role of NPCs is to trigger pathway activity. As the parenchymal component, HCs synthesize the majority of essential products (the main body of cascades/pathways), except the triggers, which may prevent the HCs from initiating uncontrolled and dangerous cascades. In addition, HCs express concentrated inhibitors that could neutralize free triggers. The nonparenchymal cells (HSCs, KCs, and LSECs) act as regulators by supplying key triggers to the system. The specificities of this system were validated by Western blotting (WB) and PRM (Fig. 3*G*). The diversity and complementarity of PCs and NPCs in the liver may represent common features of pathways implemented within the organs.

To further confirm the model of PC making/NPC triggering cascades, we analyzed upstream TFs that regulate the triggers and downstream proteins in the coagulation and complement pathways. Using the CellNet database of TFs and genes (29), we found that TFs that regulate triggers were significantly enriched in NPCs, whereas TFs that regulate downstream protein production were enriched in PCs (Fig. 3*H* and supplemental Fig. S4*A*). As an example, both the mRNA levels of C1qa/b/c, and Vwf and the protein levels of their upstream TFs were enriched in NPCs compared with PCs (Fig. 3*I*). These findings revealed that the PC production of pathway components and the NPC role in triggering the pathways are related to the differential expression of upstream TFs in different cell types.

##### Hierarchical Proteome Crosstalk Networks Among the Cell Types From Ligand-receptor to TF-TG Target the Cellular Functions of the Liver Organ

**Fig. 4.**
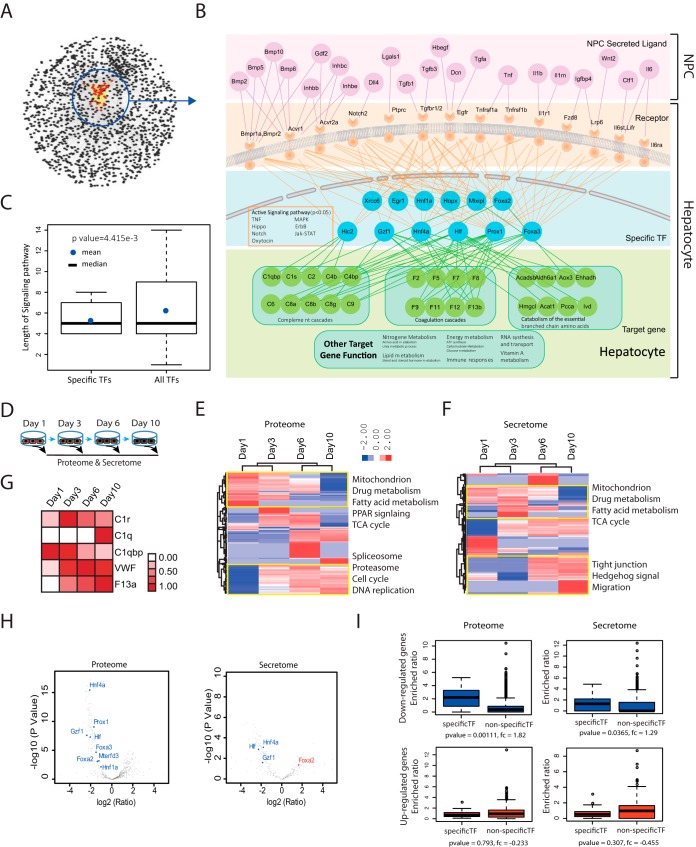
**Ligand-receptor-specific TF signaling transduction is critical in hepatocyte identity.** *A*, Theoretical ligand-receptor-TF signal transduction network shows a co-clustering of HC-specific receptors and specific TFs. *B*, Pathway nodes of ligand-receptor-specific TF-TG are significantly fewer than those of nonspecific TFs. The signaling pathways, including Notch, Mapk, Erbb, and others, are significantly activated (*p* < 0.01 according to a hypergeometric test) and significantly shorter than the signaling pathways regulating all TFs (*p* < 0.01 by Wilcoxon rank sum test). *C*, A zoomed-in network of the circled region in (*A*) illustrates the interactions and signal transductions of ligand-receptor-specific TF-TG in HCs. NPCs secreted ligands in the extracellular space to interact with receptors, specific TFs, and downstream TGs in HCs. *D*, Proteomes and secretomes of HCs cultured for 1, 3, 6, and 10 days were processed for MS identification. *E*, *F*, Hierarchical clustering shows that the proteome and secretome of cultured HCs deviated from their primary status. *G*, C1r, C1q, C1qbp, Vwf, and F13a expression profiles in HCs secretome were altered after culture compared with isolated primary HCs. *H*, *I*, Downregulated proteins in cultured HC supernatants were enriched in target gene groups of HC-specific TFs.

To understand the cellular crosstalk among the four major liver cell types involved in maintaining HC identity, we constructed a computational model for crosstalk signaling of the organ based on signal transduction and protein interactions (including ligands, receptors, TFs, and target genes (TGs)) according to CCCEXPLOR algorithms (27, 34). In this model, ligands expressed in NPCs (enhanced expression in NPCs or secreted from NPCs) and their corresponding receptors expressed in HCs were retrieved as potential crosstalk components. Activated downstream signaling pathways of the receptors in HCs were identified by analyzing expressed receptors, specific TFs and signaling nodes that connect receptors and specific TFs. The expression of specific TFs was cell-type specific, and the expression level in certain cell types was ten times larger compared with the geometric mean of the expression levels in the other cell types. This differential expression played an important role in cell identity determination. Only the significantly enriched signaling pathways (Hypergeometric test; *p* < 0.05) were combined to establish the crosstalk network. The generated network was simplified by linking receptors directly to the TFs and adding the TGs (makers in HCs) of specific TFs (Fig. 4*A*, Fig. 4*B*, and supplemental Table S4).

The derived crosstalk network demonstrated interactions among ligands from NPCs, specific receptors, specific TFs, and TGs in HCs (Fig. 4*A*, Fig. 4*B*, and Fig. 4*C*). The average shortest path length of derived crosstalk signaling pathways regulating specific TFs was significantly shorter than those regulating all TFs (5.6 *versus* 6.1, Mann-Whitney *U* test, *p* value = 4.414e-3), indicating that NPCs maintain HC identity through a fast and effective crosstalk signaling process. This phenomenon reveals the precise complementarities in the division of labor among the four liver cell types and shows that they are regulated by PC-NPC crosstalk (Fig. 4*C* and supplemental Fig. S4*B*). This complementarity was also supported by the active status of specific TFs in HCs: increased expression of positively regulated TFs for downstream components and negatively regulated TFs for triggers, but decreased expression of negatively regulated TFs for downstream components and positively regulated TFs for triggers (supplemental Fig. S4*A*).

To determine the effects of hierarchical proteome crosstalk networks of the four liver cell types in controlling the protein expression patterns of PCs, we isolated primary HCs for *in vitro* cultivation. We cultured isolated primary HCs for 1, 3, 6, and 10 days (Fig. 4*D*) to monitor the changes in the resident proteome and secretome (supplemental Table S5). As shown in Fig. 4*E* and Fig. 4*F*, the proteome and secretome of cultured HCs gradually deviated from the original HC state. Over time, the major biological functions of HCs, such as mitochondrial metabolism, fatty acid metabolism, and drug metabolism, were decreased, whereas their involvement in the cell cycle, migration, and DNA replication were elevated, indicating an identity loss and HC de-differentiation. In *ex vivo* culturing conditions, the protein specialization that occurs in the presence of other specialized cells is disrupted. Triggers, such as C1r, C1q, Vwf, and F13a, in coagulation and complementation pathways were gradually up-regulated, whereas the downstream pathway component C1qbp was downregulated (Fig. 4*G*).

We found that downregulated proteins were significantly enriched in the TG groups of the specific HC TFs (Fig. 4*H* and supplemental Table S5). This trend held true for all of the downregulated proteins (Fig. 4*I*), suggesting that HCs lose specific TFs when the HC cell fate is altered. Taken together, these data suggest that *ex vivo* culturing of primary hepatocytes leads to a rapid loss of PC identity in the absence of NPCs, suggesting that NPCs in the liver and NPC-PC crosstalk are required to maintain the identity of PCs through the activation of specific TFs by signal transduction.

In summary, cell-type-resolution liver proteomics has revealed three basic features of the liver cell types that make up the entire organ. (1) The PC (*i.e.* the HC) is the main cell of the organ, and its proteome executes most of the fundamental cellular functions for the organ. HCs express the least number of GPs and have the highest homogeneity but unique proteome patterns, although they represent 90% of all liver cells. (2) The division of labor between PCs and NPCs follows a system in which the PCs make main components of pathways, but NPCs trigger the pathways. (3) Crosstalk among NPCs and PCs maintains the PC phenotype. These features may represent general principles in the cellular composition of the organ.

## DISCUSSION

In the “-omics” age, it is desirable to determine entire suites of expressed proteins and the changes they undergo during a process of interest. The substantial improvement of next-generation proteomics (21, 35, 36) has extended its applications into wider biological fields. In-depth and precise proteomics analysis capable of systematically describing cell and tissue proteomes with high spatiotemporal resolution is both possible and highly valuable (37). As a result, many accurate proteomes of primary cell types have been reported in recent years (38, 39). Organs consist of multiple cell types that collaborate with each other to perform organ functions. Cell-type-resolved proteomics allows us to precisely dissect organ protein profiles and to understand features of cellular composition in the organ. The liver is the largest and most typical parenchymal organ in the body and is responsible for the metabolism of lipids, amino acids, and carbohydrates; synthesis of serum proteins; drug metabolism; and other functions. Interactions among four major liver cell types—HCs, HSCs, LSECs, and KCs—are critical components of liver function. We previously presented the “Liverbase” of the Human Proteome Organization (HUPO), which included 6788 proteins of the adult human liver, representing the first organ whose proteome had been investigated at the organ level (16). Very recently, S. Babak Azimifar *et al.* reported a cell-type-resolution liver proteome data resource (19). These data sets are large-scale proteome resources for studying the liver and its major cell types, but they do not provide comprehensive insight into the biology of the liver and the features of the organ's cellular composition. In this study, we employed FACS to sort liver cell types with high purity and utilized the Fast-seq approach for deep proteome coverage and accurate proteome quantification (21). A precise and reproducible cell-type-resolution liver proteome with 10,075 identified GPs was obtained, achieving over 80% coverage of RNA-Seq on the mRNA level (Fig. 1). The high consistency between specific proteome features and the physiological activities of each cell type demonstrated the validity of our data set. In addition, as the parenchymal component, the simpler HC proteome was shown to execute most of the fundamental cellular functions for the organ. The low protein identity in the HC proteome is not an accident, and protein quantity and abundance were strictly regulated, making the proteome different from the transcriptome. HCs expressed unique proteome and transcriptome patterns, representing the highest homogeneity and the most fundamental cellular functions in the liver (Fig. 2).

A systematic analysis of the proteomes of four liver cell types revealed clear divisions of labor in the metabolism, coagulation, and complement pathways. These findings indicate a novel concept in organogenesis, from the perspective of proteomics, *i.e.* the concept that PCs make the downstream components of the pathway, but NPCs trigger the pathways. This collaboration among the four cell liver types explains how the liver efficiently performs functions with precise control and demonstrates the advantage of cell-type-resolution proteomics over whole-organ proteomics in elucidating the specific functions of different cell types (Fig. 3).

TFs control almost all biological processes, ranging from cell cycle regulation to organ morphogenesis (40). We integrated the proteome analysis by employing the TF-TG network from CellNet (29) and found that the specificities and complementarities of protein expression in the PCs and NPCs were enriched in the target gene groups of their specific TFs (Fig. 3). Crosstalk among the four liver cell types contributes to the liver microenvironment (41). We found that crosstalk signaling pathways of different liver cell types form directed and connected networks of ligand-receptor interactions with specific TFs and their TGs, which were closely related to cell-type identities (Fig. 4). Primary isolated HCs, which lost PC-NPC crosstalk, underwent dedifferentiation in tissue cultures (19, 42). We found that HC features such as lipid metabolism and drug metabolism were downregulated. During the process, the target gene expression levels of hepatocyte-specific TFs changed much more dramatically than noncell-specific TFs when the cell types were profoundly altered.

Our findings suggest certain principles in the cellular composition of the liver. The PC component, HCs, represents a unique and the most homogenous proteome pattern to execute the majority of the fundamental cellular functions of the liver, whereas NPCs had more complex proteomes to govern regulatory processes. The allocation of functions between PCs and NPCs follows a model in which PCs make downstream components of the pathway, but NPCs trigger the pathways. The cell type identity and highly efficient division of labor are maintained by hierarchical proteome network crosstalk among the cell types, ranging from ligand-receptor interactions to TF-TG interactions, which target specific cellular functions.

Taken together, this study chose the liver as a model organ to measure cell-type-resolved proteomes, aiming to uncover features of the cellular proteomes to understand the division of labor and the collaboration between different cell types that compose the organ, demonstrating the feasibility of employing big data based life-omics to dissect the features and principles of organisms (43, 44). This type of approach to cell-type-resolved organ proteomics could also be applied to other organs, such as lung, stomach, and heart.

## Supplementary Material

Supplemental Data

## Footnotes

^1^ The abbreviations used are:

PC: parenchymal cell
AGC: automatic gain control
AIH: autoimmune hepatitis
DMEM: Dulbecco's modified Eagle's medium
FA: formic acid
FACS: fluorescence activated cell sorting
FDR: false discovery rate
FPKM: fragments per kilobase of exon per million fragments mapped
GO: gene ontology
GP: gene product
HC: hepatocyte
HCD: higher-energy collision dissociation
HPLC: high-performance liquid chromatography
HSC: hepatic stellate cell
iBAQ: intensity based absolute protein quantification
KC: Kupffer cell
LC-MS: liquid chromatograph-mass spectrometer
LSEC: liver sinusoidal endothelial cell
MACS: magnetic-activated cell sorting
PRM: parallel reaction monitoring
MS: mass spectrometry
NASH: nonalcoholic steatohepatitis
NPC: nonparenchymal cell
RPLC: reversed-phase liquid chromatography
TF: transcription factor
TG: target gene.
