# Supplementary material for: A Cell-type-resolved Liver Proteome
Source: Mol Cell Proteomics. 2016 Aug 25;15(10):3190–202. doi: 10.1074/mcp.M116.060145 (PMC5054343; doi:10.1074/mcp.M116.060145)
Supplement: Supplemental Data [file supp_15_10_3190__index.html]

A cell-type-resolved liver proteome — A Cell-type-resolved Liver Proteome — Cell-type-resolved Liver Proteome — Supplemental Data 

# A Cell-type-resolved Liver Proteome

## Supplemental Data

- Supplementary Table 1 (.xlsx, 5.8 MB) - Expression level of gene in 4 cell types in proteome and transcriptome level.
- Supplementary Table 1 (.xlsx, 277 KB) - Gene Ontology (GO) enrichments of over- and under- represented proteins in the detected proteome compared to the transcriptome. Expressions of HC, HSC, KC, LSEC, HC secretome as well as KC secretome are compared to the corresponding transcriptomes. Both over- and under- represented proteins are annotated by DAVID Bioinformatics Resources.
- Supplementary Table 3 (.xlsx, 136 KB) - GO/pathway/disease enrichments of gene products, including only detected in the proteome and genes only existed in transcriptome. GO are annotated by DAVID Bioinformatics Resources.
- Supplementary Table 4 (.xlsx, 137 KB) - Enrichment of Transcription Factors (TF) and Target Genes (TG) in 4 cell types and ligand - receptor pairs in 4 cell types . Enrichment is measured by z-score.
- Supplementary Table 5 (.xlsx, 1.0 MB) - The changes in the resident proteome and secretome and the GO enrichments of secretome/wce specific proteins. Expression changes of HC 1, 3, 6 and 10 days of WCE and secretome are annotated by DAVID Bioinformatics Resources.
- Supplemental Text (.pdf, 88 KB) - Supplemental Text
- Supplemental Figures (.pdf, 27.0 MB) - Supplemental Figures and Supplemental Table Legend.
