# Supplementary material for: A Cell-type-resolved Liver Proteome
Source: Mol Cell Proteomics. 2016 Aug 25;15(10):3190–202. doi: 10.1074/mcp.M116.060145 (PMC5054343; doi:10.1074/mcp.M116.060145)
Supplement: Supplemental Data [file 10.1074_M116.060145_mcp.M116.060145-6.pdf]

Supplemental Text

Protein identification and quantification using MaxQuant

In order to compare the effect of protein identification with or without matching between runs, we also performed MaxQuant search for our dataset. Specifically, Raw files were searched with MaxQuant 1.5.3.30 search engine against mouse RefSeq protein database (29,764 proteins, updated on 07-01-2013). Trypsin was used to generate peptides. A target-decoy-based strategy was applied to control both peptide- and protein -level false discovery rates (FDRs) lower than 1%. The fixed modification was carbamidomethyl (C), and the variable modifications were oxidation (M) and acetyl (Protein N-term). The special amino acids were Lys (K) and Arg (R). The Orbitrap MS tolerance of precursor ions was 20ppm, and 20ppm for fragment ions. The minimum peptide length was 7, and the minimum score for modified peptides was 40. And maximum 2 cleavages were permitted for the number of missed and/or non-specific cleavages. We performed the data searching both with and without match between runs to get the comparison. For match between runs, the matching time window was 0.7 minute and the alignment time window was 20 minutes. Other parameters were the standard parameters of MaxQuant. The abundances for all proteins were estimated by applying iBAQ algorithm, and then normalized to FOT (a fraction of the total protein iBAQ amount per experiment).

Supplemental figure legends

Figure S1. Isolation of 4 major liver cell types.

(A-D) Evaluation of cell purity, viability and yield. (A, D) Using the modified method for cell isolation and validation, the cell yields of the HCs, HSCs, KCs and LSECs were approximately (7.0 ±0.4) x 10^7, (1.1 ±0.2) x 10^6, (2.1 ±0.2) x 10^6, and (2.1 ±0.2) x 10^6 per mouse, respectively. (B) The viabilities of the HCs, HSCs, KCs and LSECs were determined by Trypan blue staining and 7-AAD flow cytometry and were as follows: (90.6 ±0.7)%, (88.3 ±0.5)%, (88.4 ±0.5)%, and (87.3 ±0.3)%, respectively. (A, C) Various evidence using bright microscopy, electron microscopy, autofluorescence testing, immunocytochemistry, and FACS analysis confirmed that the purities of the HCs, HSCs, KCs and LSECs were (98.6 ±0.5)%, (93.7 ±0.4)%, (94.6 ±0.2)%, and (98.0 ±0.5)%,

respectively. (E) Protein quality was validated by gel electrophoresis (SDS-PAGE). After protein extraction from each cell type, gel electrophoresis of the whole-cell extract was performed with a 12% separating gel and a 5% stacking gel at 80 V for 20 min and 120 V for 60 min in the buffer system. Coomassie brilliant blue staining was used to determine protein bands in all samples.

Figure S2. Proteomes and transcriptomes of 4 major liver cell types

(A-C) Comparison and correlation of the 4 cell types in 3 replicates at the protein and peptide levels, separately. (A) The overlaps of protein and peptide identifications ranged from 80%-90% in at least 2 of 3 replicates, as shown in the Venn diagram. (B) The correlation coefficients for HCs, HSCs, KCs and LSECs in 3 replicates were approximately 0.867, 0.845, 0.864 and 0.879 at the protein level, respectively, and 0.741, 0.636, 0.686 and 0.687 at the peptide level on average, respectively. (C) Comparisons of abundance profiles (fraction of the total) and CV (coefficient of variation for protein expression in four liver cell samples) of three classes of proteins (detected three times in three replicates). The differences among the three classes are statistically significant (Mann-Whitney U Test, $p<0.05$). (D-J) Comparison of the proteome and transcriptome. (D) The identification comparison and (E) the correlation coefficient of the total proteome and transcriptome identified in the 4 cell types was approximately 0.50. (F) Genes were identified exclusively in the proteome and transcriptome in the 4 cell types; a total of 264 and 1212 gene products were exclusively identified in the proteome and transcriptome datasets, respectively. (G) Density scatter plot of iBAQ intensities of proteome versus FPKM values of the transcriptome in 4 liver cell types. The color code indicates the percentage of points that are included in a region of a specific color. A total of 95% of the dots were in the space between the red and green lines. The genes on the red line were overrepresented in the proteome, and the genes below the green line were overrepresented in the transcriptome. (H) Comparison of the transcriptome and the resident proteome plus secretome in the HCs and KCs is shown. (I) Venn diagram of the identification of the transcriptome, proteome and secretome of the HCs. (J) GO enrichments of the proteome and the transcriptome overrepresented genes in 4 cell types. The significance of gene enrichment of the extracellular space in the transcriptome compared to the proteome or proteome plus secretome is shown.

Figure S3. Differential proteome patterns of 4 cell types

(A) Hierarchical clustering of liver, NPC, HC, HSC, KC, and LSEC proteomes shows that liver and HC proteomes were co-clustered, while HSC, KC, and LSEC proteomes were co-clustered with the NPC proteome. (B) Top: Chromosomal coverage of the 10,506 gene products in each cell identified in this study. Bottom: Average chromosomal expression level of gene products in each cell identified in this study. (C) Coverage and protein expression levels in three categories of GO items (BP, biological process; CC, cellular component; MF, molecular function). The 2nd level GO terms belong to the corresponding 1st level, and the 3rd level terms belong to the corresponding 2nd level. (D) Relative protein expression levels (z-scores) of liver disease-related genes in each cell identified in our study. The genes related to liver diseases were retrieved from the LoMA database. The difference between cell types was statistically significant and was determined using the Mann-Whitney U test.

Figure S4. Crosstalk between HCs and NPCs

(A) The positive regulatory TFs of "makers" were overexpressed in PCs, while the positive regulatory TFs of "triggers" were overexpressed in NPCs. Compared to the NPCs, triggers in the HCs were repressed because of the depression of positive regulatory TFs, whereas makers in the HCs were activated because of the elevation of positive regulatory TFs and the depression of negative regulatory TFs. The TF-TG relationship and direction were retrieved from CellNet. (B) Crosstalk network of ligand receptor-specific TF-TG in NPCs. We combined the active signaling pathways of the HSCs, KCs, LSECs into the NPCs. Ligands secreted by PCs bound to receptors in the NPCs, and downstream signaling pathways were activated. The TFs that regulated triggers were regulated by these signaling pathways.
