# Supplementary material for: A Cell-type-resolved Liver Proteome
Source: Mol Cell Proteomics. 2016 Aug 25;15(10):3190–202. doi: 10.1074/mcp.M116.060145 (PMC5054343; doi:10.1074/mcp.M116.060145)
Supplement: Supplemental Data [file 10.1074_M116.060145_mcp.M116.060145-7.pdf]

Supplemental Information

A cell-type-resolved liver proteome

Chen Ding[1, 2, 5]*, Yanyan Li[3]*, Feifei Guo[1, 2]*, Ying Jiang[1, 2]*, Wantao Ying[1, 2]*, Dong Li[1, 2]*, Dong Yang[1, 2], Xia Xia[1, 2], Wanlin Liu[1, 2], Yan Zhao[1, 2], Yangzhige He[1, 2, 3], Xianyu Li[1, 2], Wei Sun[1, 2], Qiongming Liu[1, 2], Lei Song[1, 2], Bei Zhen[1, 2], Pumin Zhang[1, 2], Xiaohong Qian[1, 2#], Jun Qin[1, 2, 4, 5#], and Fuchu He[1, 2, 5#]

[1]State Key Laboratory of Proteomics, Beijing Proteome Research Center, Beijing Institute of Radiation Medicine, Beijing 100039, China; [2]National Center for Protein Sciences (The PHOENIX center, Beijing), Beijing 102206, China; [3]School of Life Sciences, Tsinghua University, Beijing 100084, China; [4]Alkek Center for Molecular Discovery, Verna and Marrs McLean Department of Biochemistry and Molecular Biology, Department of Molecular and Cellular Biology, Baylor College of Medicine, Houston, Texas 77030, USA; [5]State Key Laboratory of Genetic Engineering and Collaborative Innovation Center for Genetics and Development, School of Life Sciences, Institute of Biomedical Sciences, Fudan University, Shanghai 200433, China.

*Equal contributing authors

[#]To whom correspondence should be addressed: hefc@nic.bmi.ac.cn (F.H.); jqin@bcm.edu (J.Q.); qianxh1@163.com (X.Q.)

Ding et al, Supplemental Information
Supplemental Figures and Figure Legends

Figure S1

A

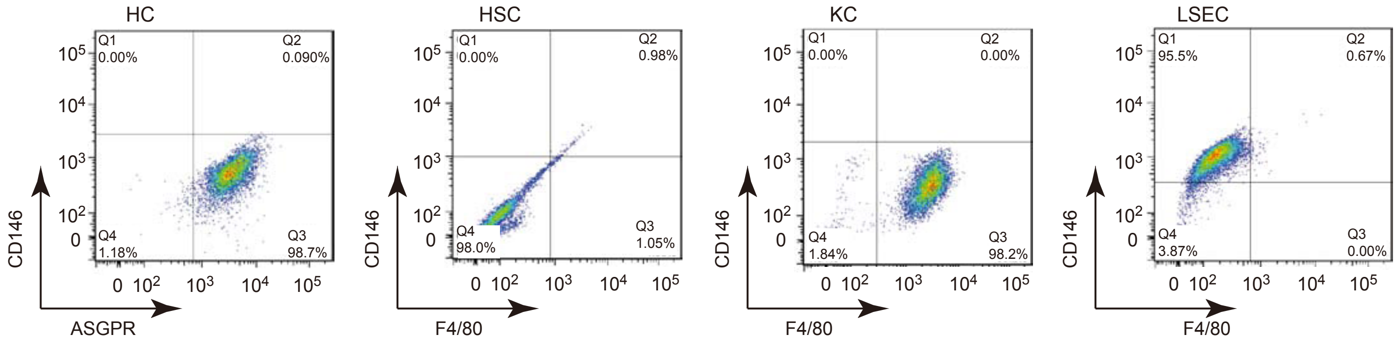

B

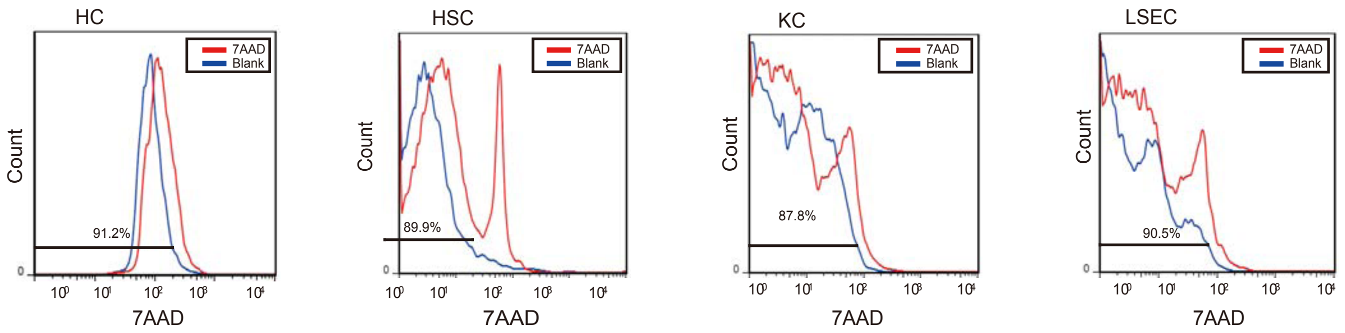

C

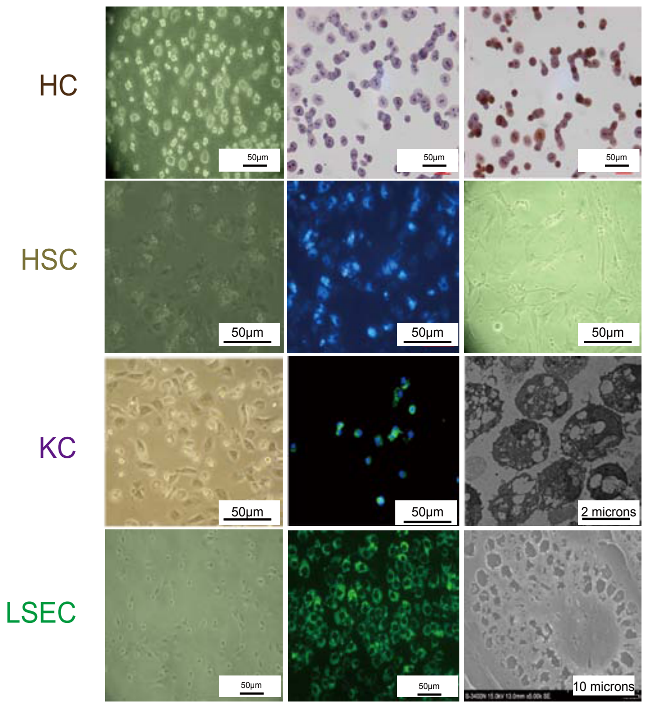

D

Cell	Yield/liver	Viability	Purity
HC	(7.0± 0.4)*10^7	90.6± 0.7%	98.6± 0.5%
HSC	(1.1± 0.2)*10^6	88.3± 0.5%	93.7± 0.4%
KC	(2.1± 0.2)*10^6	88.4± 0.5%	94.6 ± 0.2%
LSEC	(2.1± 0.2)*10^6	87.3± 0.3%	98.0± 0.5%

E

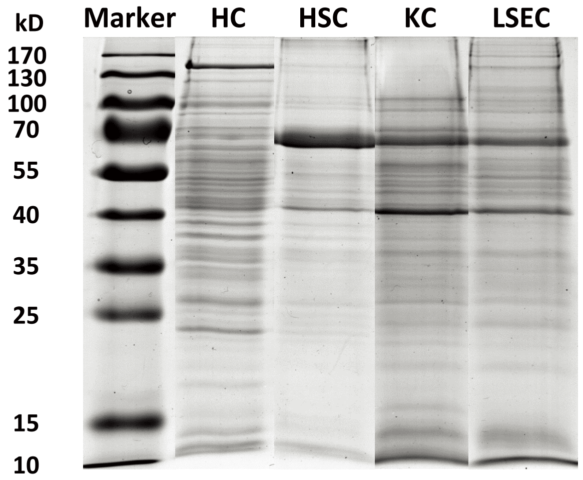

Figure S1. Isolation of 4 major liver cell types.

(A-D) Evaluation of cell purity, viability and yield. (A, D) By the modified method for cell isolation and validation, cell yields of HCs, HSCs, KCs and LSECs were about $(7.0\pm0.4)*10^7$, $(1.1\pm0.2)*10^6$, $(2.1\pm0.2)*10^6$, and $(2.1\pm0.2)*10^6$ per mouse, respectively. (B) Viabilities of HCs, HSCs, KCs and LSECs were (90.6±0.7)%, (88.3±0.5)%, (88.4±0.5)%, and (87.3±0.3)%, evaluated with trypan blue staining and 7-AAD flow cytometry. (A, C) Various evidences of bright microscopy, electron microscopy, autofluorescence test, immunocytochemistry, and FACS analysis have confirmed that purities of HCs, HSCs, KCs and LSECs were (98.6±0.5)%, (93.7±0.4)%, (94.6±0.2)%, and (98.0±0.5)%, respectively. (E) Protein quality validated by gel electrophoresis of SDS-PAGE. After protein extraction from each cell type, gel electrophoresis of whole cell extract was performed with 12% separating gel and 5% stacking gel, 80V voltage for 20min and 120V voltage for 60min in the buffer system. Coomassie brilliant blue staining was used to determine protein bands in all samples.

Figure S2A-C

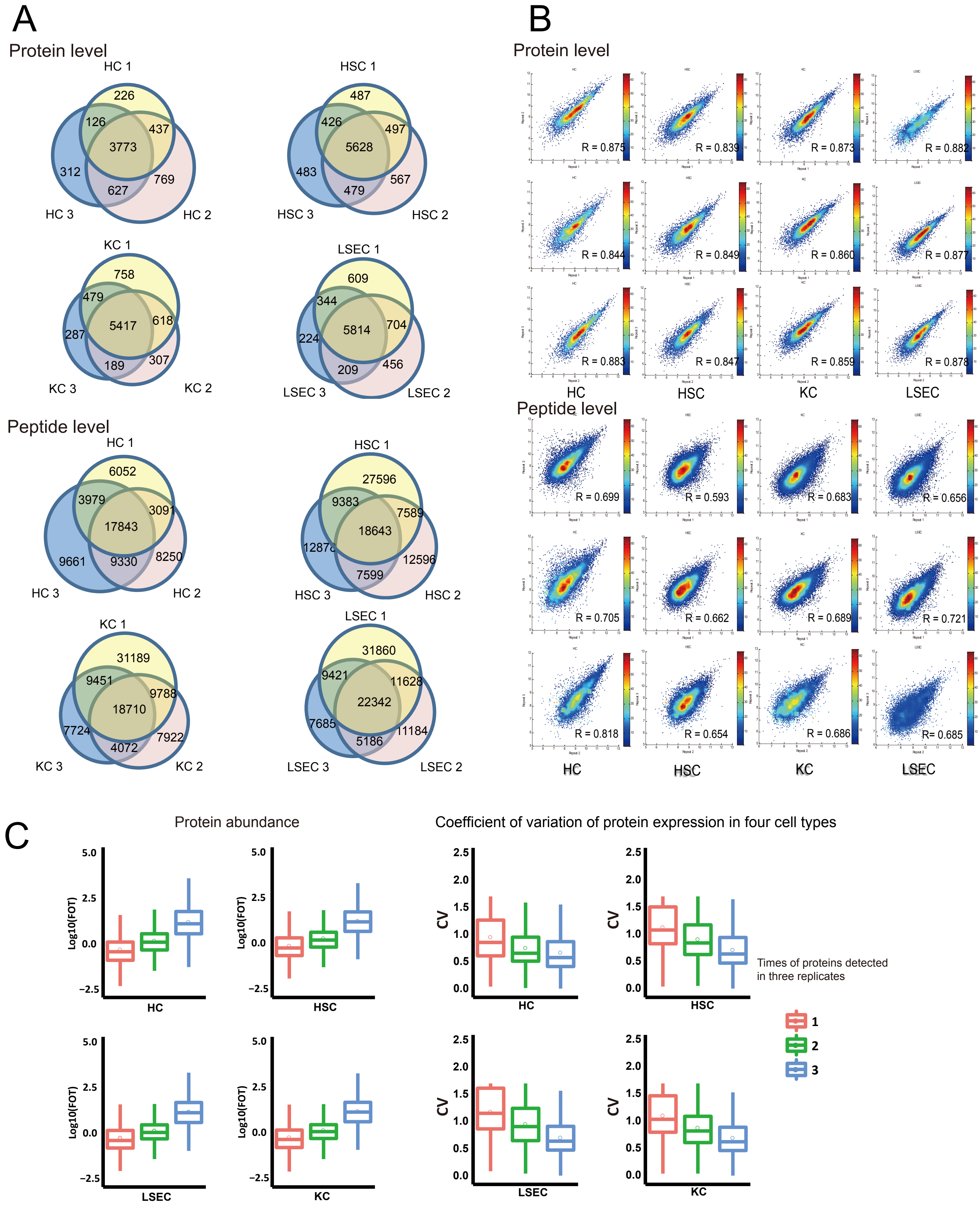
A
Protein level
HC 1
226
126
437
3773
312
769
627
HC 3
HC 2
HSC 1
487
426
497
5628
483
567
479
HSC 3
HSC 2
KC 1
758
479
5417
618
287
189
307
KC 3
KC 2
LSEC 1
609
344
5814
704
224
209
456
LSEC 3
LSEC 2
Peptide level
HC 1
6052
3979
3091
17843
9661
9330
8250
HC 3
HC 2
HSC 1
27596
9383
7589
18643
12596
7599
HSC 3
HSC 2
KC 1
31189
9451
9788
18710
7724
7922
4072
KC 3
KC 2
LSEC 1
31860
9421
11628
22342
7685
11184
5186
LSEC 3
LSEC 2
B
Protein level
R = 0.875
R = 0.839
R = 0.873
R = 0.882
R = 0.844
R = 0.849
R = 0.860
R = 0.877
R = 0.883
R = 0.847
R = 0.859
R = 0.878
HC
HSC
KC
LSEC
Peptide level
R = 0.699
R = 0.593
R = 0.683
R = 0.656
R = 0.705
R = 0.662
R = 0.689
R = 0.721
R = 0.818
R = 0.654
R = 0.686
R= 0.685
HC
HSC
KC
LSEC
C
Protein abundance
Coefficient of variation of protein expression in four cell types
Log10(FOT)
CV
HC
HSC
LSEC
KC
Times of proteins detected in three replicates
1
2
3

Figure S2D-J

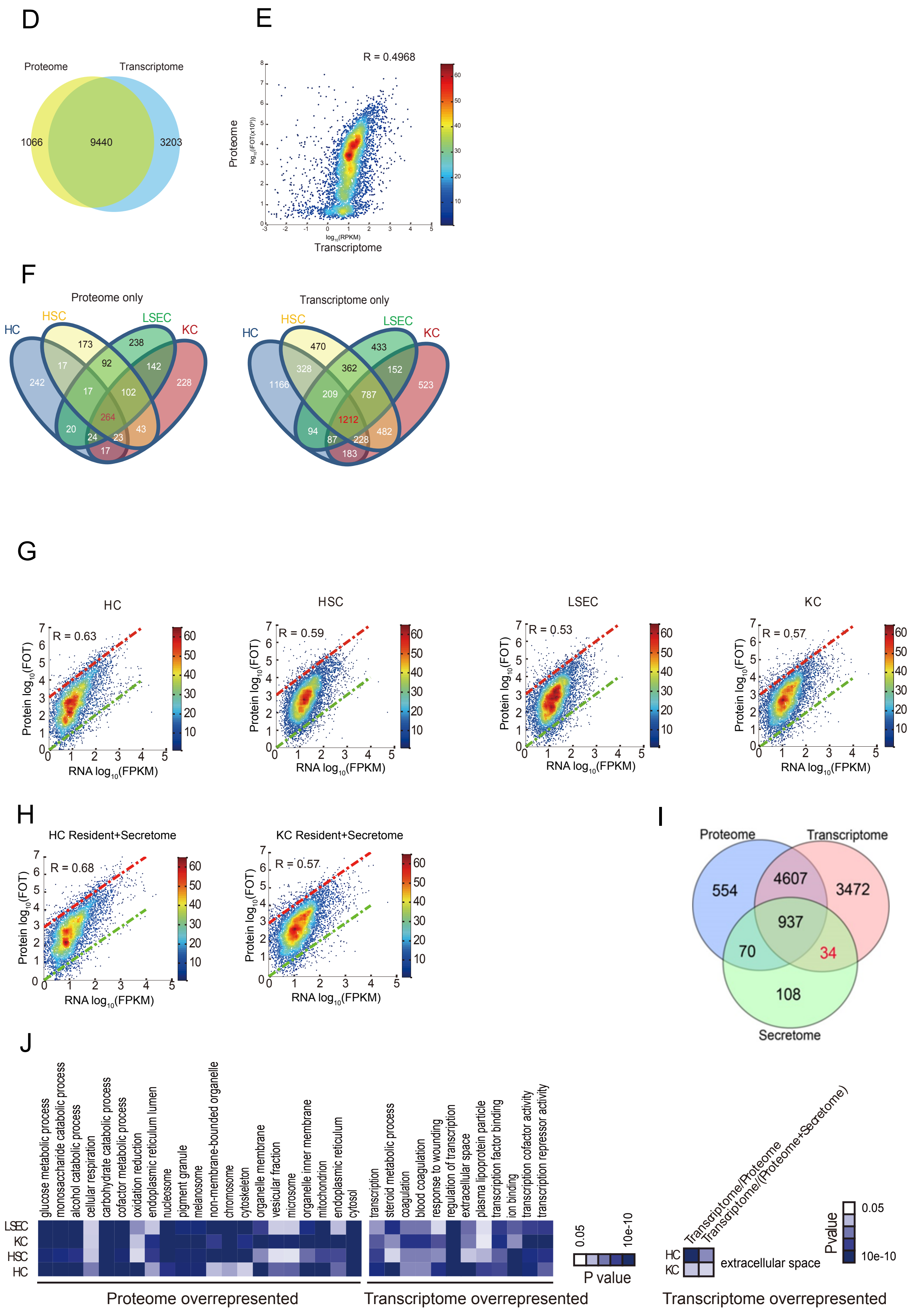
D
Proteome
Transcriptome
1066
9440
3203
E
R = 0.4968
Proteome
Transcriptome
F
Proteome only
Transcriptome only
HC
HSC
LSEC
KC
G
HC
HSC
LSEC
KC
R = 0.63
R = 0.59
R = 0.53
R = 0.57
Protein log10(FOT)
RNA log10(FPKM)
H
HC Resident+Secretome
KC Resident+Secretome
R = 0.68
R = 0.57
I
Proteome
Transcriptome
Secretome
554
4607
3472
937
70
34
108
J
glucose metabolic process
monosaccharide catabolic process
alcohol catabolic process
cellular respiration
carbohydrate catabolic process
cofactor metabolic process
oxidation reduction
endoplasmic reticulum lumen
nucleosome
pigment granule
melanosome
non-membrane-bounded organelle
chromosome
cytoskeleton
organelle membrane
vesicular fraction
microsome
organelle inner membrane
mitochondrion
endoplasmic reticulum
cytosol
transcription
steroid metabolic process
coagulation
blood coagulation
response to wounding
regulation of transcription
extracellular space
plasma lipoprotein particle
transcription factor binding
ion binding
transcription cofactor activity
transcription repressor activity
LSEC
KC
HSC
HC
0.05
10e-10
P value
Proteome overrepresented
Transcriptome overrepresented
Transcriptome/Proteome
Transcriptome/(Proteome+Secretome)
extracellular space
Pvalue
Transcriptome overrepresented

Figure S2. Proteomes and transcriptomes of 4 major liver cell types.

(A-C) Comparison and correlation of the 4 cell types in 3 replicates at the protein and peptide level, separately. (A) The overlap of protein and peptide identifications was up to 80%-90% in at least 2 of 3 replicates, as shown in Venn diagram. (B) Correlation coefficients of HC, HSC, KC and LSEC in 3 replicates were about 0.867, 0.845, 0.864 and 0.879 at the protein level and 0.741, 0.636, 0.686 and 0.687 at the peptide level in average, respectively.(C) Comparison of abundance(fraction of total) and CV (Coefficient of variation for protein expression in four liver cell samples) of three classes proteins (detected once, twice and thrice in three replicates). Differences between three classes are statistical significant (Mann-Whitney U Test, $p<0.05$). (D-J) Comparison of proteome and transcritome. (D) Identification comparison and (E) Correlation coefficient of the total proteome and transcriptome identified in the 4 cell types was about 0.50. (F Genes identified exclusively in the proteome and transcriptome in the 4 cell types. A total of 264 and 1212 gene products were exclusively identified in the proteome and transcriptome datasets, respectively. (G) A density scatter plot of iBAQ intensities of proteome versus FPKM values of transcriptome in 4 liver cell types. The color code indicates the percentage of points that are included in a region of a specific color. 95% dots were in the space between red and green line. Genes upon red line are overrepresented in the proteome and those below green line are overrepresented in the transcriptome. (H) Comparison of the transcriptome and the resident proteome plus secretome in HCs and KCs. (I) Venn diagram of the identification of the transcriptome, proteome and secretome of HC. (J) GO enrichment of proteome overrepresented and transcriptome overrepresented genes in 4 cell types. Significance of gene enrichment of “extracellular space in transcriptome compared to proteome or proteome plus secretome.

Figure S3

A

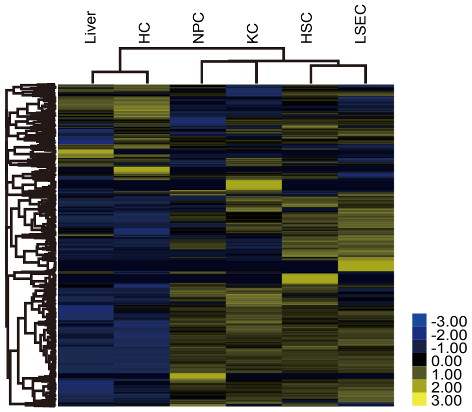

B

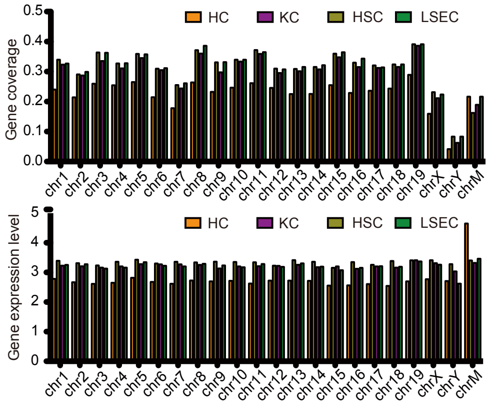

C

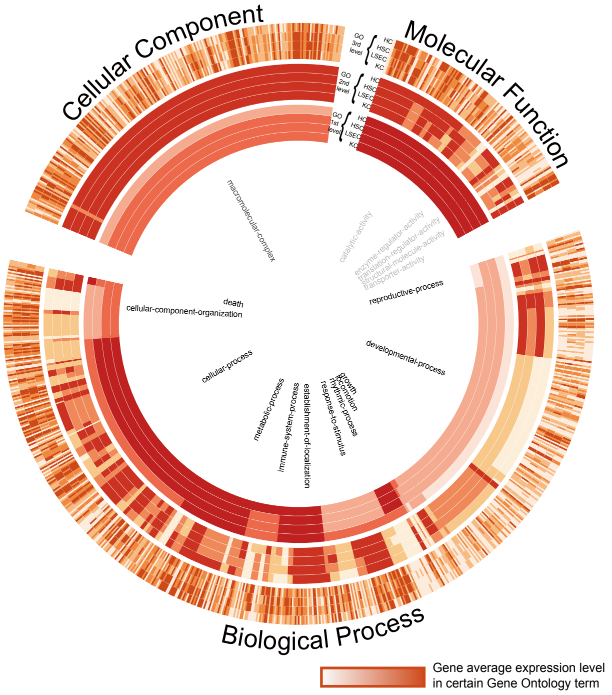

D

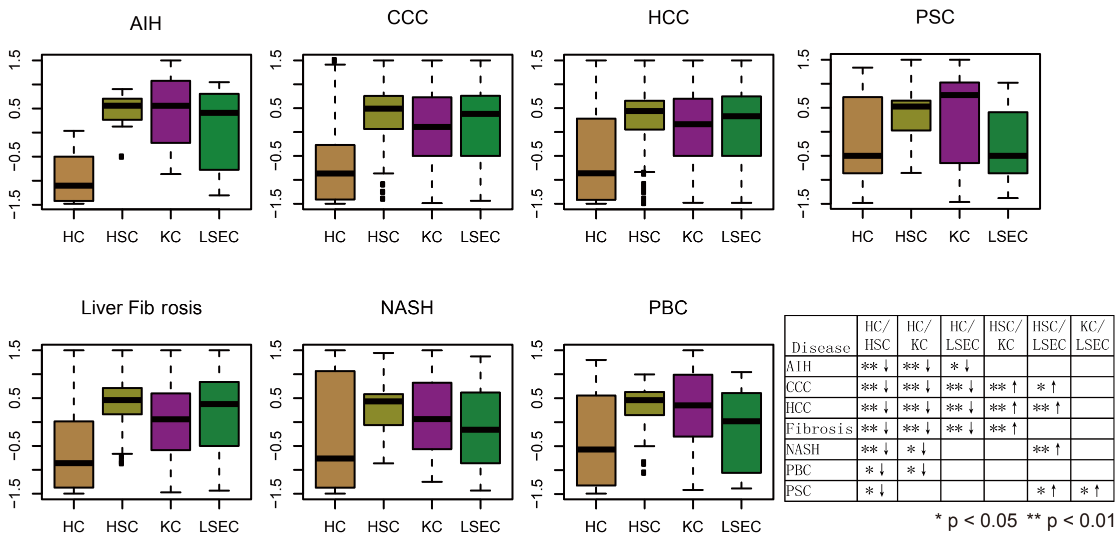

Disease	HC/ HSC	HC/ KC	HC/ LSEC	HSC/ KC	HSC/ LSEC	KC/ LSEC
AIH	** ↓	** ↓	* ↓			
CCC	** ↓	** ↓	** ↓	** ↑	* ↑	
HCC	** ↓	** ↓	** ↓	** ↑	** ↑	
Fibrosis	** ↓	** ↓	** ↓	** ↑		
NASH	** ↓	* ↓			** ↑	
PBC	* ↓	* ↓				
PSC	* ↓				* ↑	* ↑

* $p < 0.05$ ** $p < 0.01$

Figure S3. Differential proteome patterns of 4 cell types.

(A) Hierarchical clustering of liver, NPC, HC, HSC, KC, and LSEC proteomes shows that liver and HC proteomes are co-clustered, while HSC, KC, and LSEC were co-clustered with NPC. (B) Up: Chromosomal coverage of the 10,506 gene products in each cell identified in this study. Down: Average chromosomal expression level of gene products in each cell identified in this study. (C) Coverage and protein expression level on three categories of GO items (BP, biological process; CC, cellular component; MF, molecular function). 2nd level GO terms belong to corresponding 1st level, and 3rd level terms belong to corresponding 2nd level. (D) Relative protein expression level (z-score) of liver disease-related genes in each cell identified in our study. Genes related liver diseases were retrieved from LoMA database. The difference between cell types was statistical significant by Mann-Whitney U test.

Figure S4

A

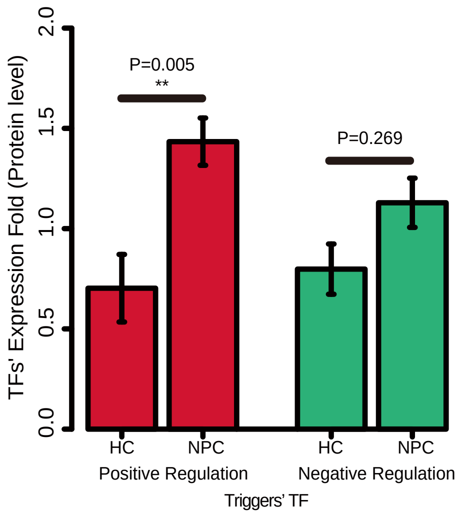

TFs' Expression Fold (Protein level)
0.0
0.5
1.0
1.5
2.0
P=0.005
**
P=0.269
HC
NPC
HC
NPC
Positive Regulation
Negative Regulation
Triggers' TF

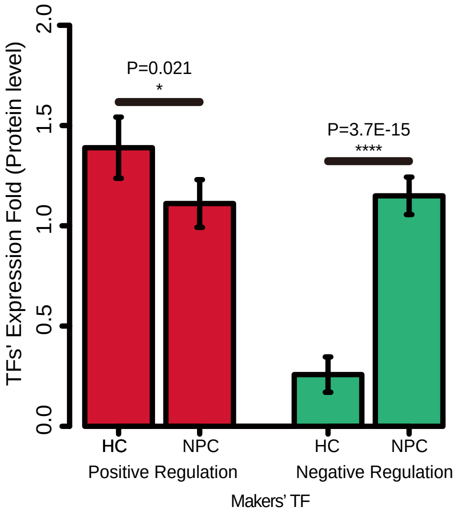

TFs' Expression Fold (Protein level)
0.0
0.5
1.0
1.5
2.0
P=0.021
*
P=3.7E-15
****
HC
NPC
HC
NPC
Positive Regulation
Negative Regulation
Makers' TF

B

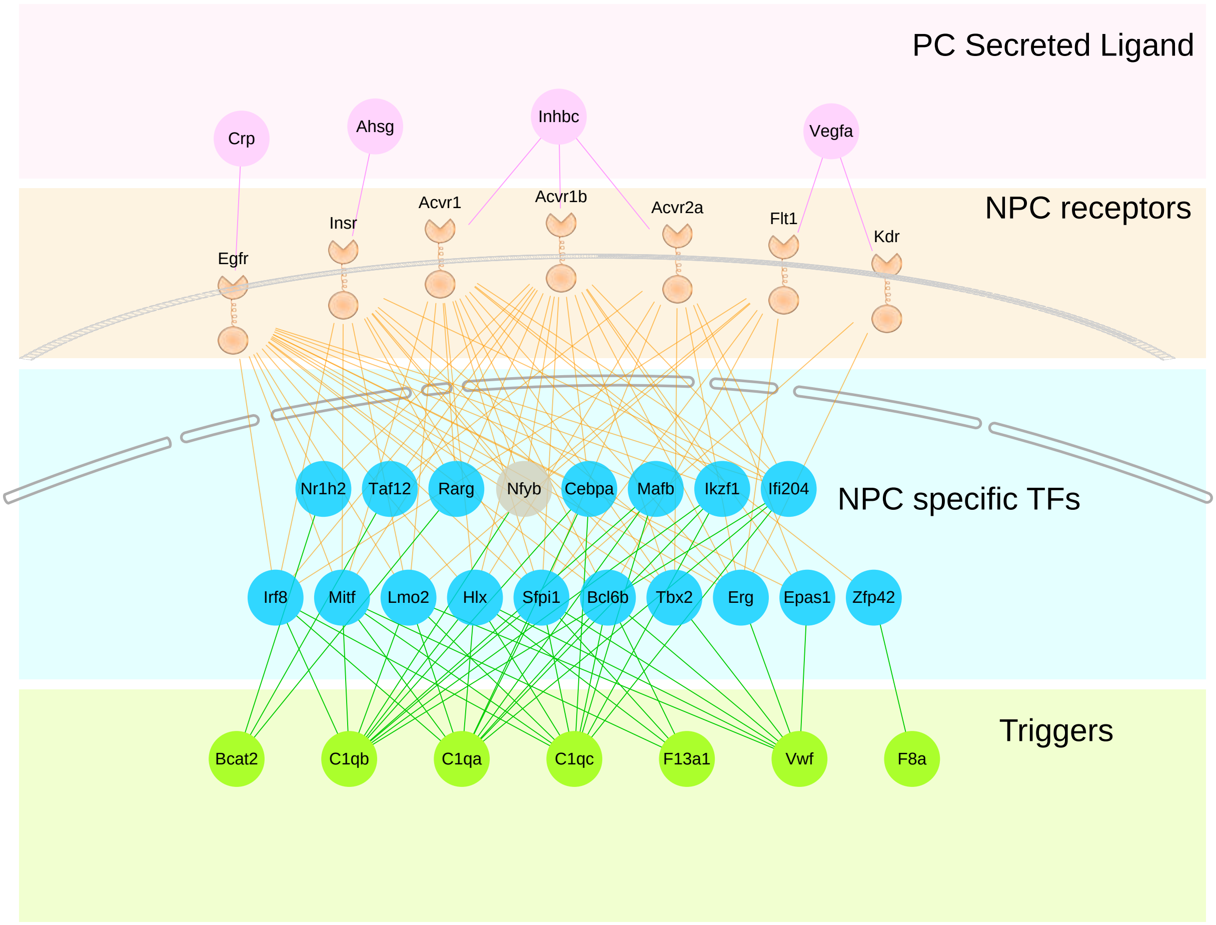

PC Secreted Ligand
Crp
Ahsg
Inhbc
Vegfa
NPC receptors
Egfr
Insr
Acvr1
Acvr1b
Acvr2a
Flt1
Kdr
NPC specific TFs
Nr1h2
Taf12
Rarg
Nfyb
Cebpa
Mafb
Ikzf1
Ifi204
Irf8
Mitf
Lmo2
Hlx
Sfpi1
Bcl6b
Tbx2
Erg
Epas1
Zfp42
Triggers
Bcat2
C1qb
C1qa
C1qc
F13a1
Vwf
F8a

Figure S4. Crosstalk between HC and NPCs.

(A) Positive regulation TFs of “makers” were overexpressing in PCs, while positive regulatory TFs of “triggers” were overexpressing in NPCs. Comparing NPC, triggers in HC were repressed because of depression of positive regulation TFs. Whereas, makers in HC were activated because of elevation of positive regulation TFs and depression of negative regulation TFs. TF-TG relation and direction were retrieved from CellNet. (B) Crosstalk network of ligand-receptor specific TF-TG in NPCs. We combine active signaling pathway of HSC, KC, LSEC into NPC. Ligands secreted by PC were binding to receptors in NPC and downstream signaling pathways were activated. TFs regulating triggers were regulated by these signaling pathways.

Table S1 Expression level of gene in 4 cell types in proteome and transcriptome level.

Table S1A. Expression level of gene products in 4 cell types. Each cell type has three biological repeats, and expression levels are represented by FOT (fraction of total). Ave means the average of FOT of three repeats. N/A means it is not detected in this condition.

Table S1B. Expression level of gene products in HC and KC secretome. Gene products located in extracellular space are marked in the "Extracellular" column**.**

Table S1C. RNA-Seq profiling in 4 cell types. RNA-Seq profiling with more than 1 fragment per kilobase of exon per million fragments mapped (FPKM). Gene products detected in proteome are marked in the "Found in Proteome" column.

Table S2. Gene Ontology (GO) enrichments of over- and under-represented proteins in the detected proteome compared to the transcriptome. Expressions of HC, HSC, KC, LSEC, HC secretome as well as KC secretome are compared to the corresponding transcriptomes. Both over- and under-represented proteins are annotated by DAVID Bioinformatics Resources.

Table S3. GO/pathway/disease enrichments of gene products, including only detected in the proteome and genes only existed in transcriptome. GO are annotated by DAVID Bioinformatics Resources.

Table S4. Enrichment of Transcription Factors(TF) and Target Genes(TG) in 4 cell types and ligand-receptor pairs in 4 cell types. Enrichment is measured by z-score.

Table S5. The changes in the resident proteome and secretome and the GO enrichments of secretome/wce specific proteins. Expression changes of HC 1, 3, 6 and 10 days of WCE and secretome are annotated by DAVID Bioinformatics Resources.
